# Sustainable Supply Chain Design: A Configurational Approach

**DOI:** 10.1155/2014/897121

**Published:** 2014-01-12

**Authors:** S. Maryam Masoumik, Salwa Hanim Abdul-Rashid, Ezutah Udoncy Olugu, Raja Ariffin Raja Ghazilla

**Affiliations:** Department of Mechanical Engineering, Centre for Product Design and Manufacturing (CPDM), Faculty of Engineering, University of Malaya, 50603 Kuala Lumpur, Wilayah Persekutuan, Malaysia

## Abstract

Designing the right supply chain that meets the requirements of sustainable development is a significant challenge. Although there are a considerable number of studies on issues relating to sustainable supply chain design (SSCD) in terms of designing the practices, processes, and structures, they have rarely demonstrated how these components can be aligned to form an effective sustainable supply chain (SSC). Considering this gap in the literature, this study adopts the configurational approach to develop a conceptual framework that could configure the components of a SSC. In this respect, a process-oriented approach is utilized to classify and harmonize the design components. A natural-resource-based view (NRBV) is adopted to determine the central theme to align the design components around. The proposed framework presents three types of SSC, namely, efficient SSC, innovative SSC, and reputed SSC. The study culminates with recommendations concerning the direction for future research.

## 1. Introduction

In recent years, increased pressure from various stakeholders, such as customers, suppliers, regulators, competitors, local and global communities, and nongovernmental organizations (NGOs), have prompted the manufacturing industry to integrate sustainability-conscious practices into their business not only at the firm level, but also for the entire supply chain [1, 2]. This shift from local optimization at the firm level towards the entire supply chain, involving the management of product flows from the initial sources of raw material to the end-user customers in both forward and reverse directions, requires a broader development of sustainability [3].

A number of studies have been conducted to investigate sustainable supply chain (SSC) practices, their drivers, and their impact on organizational performance and competitiveness. There are also studies dedicated to optimizing planning processes and designing the networks for green, reverse, and closed loop supply chains.  Table 1 shows a comprehensive list of these types of study. However, with the exception of a few research works (e.g., [4–6]), studies addressing the issues relating to alignment between SSC practices, processes, and structures are rarely found in the literature.

The concept of alignment between the design components, also called the configurational approach [7, 8], originated from the literature on organizational design and strategic management. The configurational approach in organizational design focuses on the alignment between different components of design, such as strategy, process, and structure. Meyer et al. [8] used the term *organizational configuration* for multidimensional constellations of conceptually distinct characteristics that commonly occur together. Miller [7] described configuration as a tool for creating internal harmony between the elements of an organization, which include strategy, structure, and context. He also mentioned that a central theme is needed to create such harmony.

This approach was also discussed in the literature for designing a traditional supply chain (TSC). As a case in point, Vonderembse et al. [9] described different aspects of design including organizational structure, approach to choosing suppliers, demand patterns, inventory strategy, lead time focus, manufacturing focus, product design strategy, and human resources. They suggested an optimum configuration for the supply chain with respect to the product type and life cycle, namely, lean, agile, and leagile. Chandra and Grabis [10] also discussed the supply chain as a configurable system that needs to be adapted with changes in products, processes, resources, suppliers, demand patterns, lead times, and commitment of the supply chain's echelons. More recently, Stavrulaki and Davis [11] discussed how products should be aligned with supply chain processes and strategies to increase competitive advantage. The origin of this approach can be found in the work of Fisher [12], which presented the idea of designing the right supply chain by considering the product type.

Despite the importance of alignment in achieving sustainable performance [13], which has been considered in the traditional supply chain and the strategic management literature, this particular area of research has received little attention in SSC studies. Thus, the aim of this paper is to develop a conceptual framework in which designing an aligned SSC can be achieved. The authors will initially present a better understanding of different components of sustainable supply chain design (SSCD) including SSC practices, structures and processes, and their characteristics. Subsequently, a conceptual framework for configuring these components will be developed. This framework will assist supply chain designers to design an aligned SSC and to provide a new viewpoint for academics to develop this research field further.

To develop the conceptual framework, a process-oriented approach is utilized to provide a comprehensive view for identifying and harmonizing the categorization of SSC practices, processes, and structures. A natural-resource-based view (NRBV) [14, 15] is also used to determine the central theme for these components to align around. According to the NRBV, the central theme can be “cost and risk reduction”, “innovation and repositioning”, or “reputation and legitimacy”. These are the values created through implementing the various environmental strategies.

The remainder of this paper is divided as follows: firstly, a brief definition of a SSC, a key term in this study, is provided in Section 2. This is followed by a description of the research process in Section 3. A review of the literature on SSC practices, processes, and structures is discussed in Section 4. The categorized lists of different practices, processes, and structures are also derived in this section by applying a process-oriented approach. In Section 5, a conceptual framework for configuring these components around a central theme is developed by using a configurational approach. Finally, Section 6 concludes the paper by highlighting the significant findings and recommends the direction for future development of this research.

## 2. The Concept of SSC

This section attempts to provide a comprehensive understanding concerning the concept of a sustainable supply chain (SSC) by considering the related definitions presented in the literature. A SSC consists of two key terms, namelym, “supply chain” and “sustainability”. According to Mentzer et al. [16, pp. 4], a supply chain can be defined as follows: “*a set of three or more entities (organizations or individuals) directly involved in the upstream and downstream flows of products, services, finances, and/or information from a source to a customer.*” This definition of a supply chain refers to the forward supply chain.

For the second key term—sustainability—the definition provided by Carter and Rogers [17, pp. 364] for “organizational sustainability” states that “*organizational sustainability consists of three components: the natural environment, society, and economic performance and at the intersection of these triple bottom of line, there are activities that organizations can engage in which not only positively affect the natural environment and society, but which also result in long-term economic benefits and competitive advantage for the firm.*”

In the initial period in which the concept of sustainable development was first introduced [18], most studies were dedicated to integrating environmentally friendly issues into the supply chain management. Thus, terms such as green supply chain (GSC) [19], reverse logistics (RL) [20], and closed-loop supply chain [21] were in common use. Considering the definitions of these terms would help to better understand the concept of a SSC. The following paragraphs present the definitions for these three main key terms.

Adding the term “green” to supply chain management seeks to incorporate environmentally conscious thinking in all processes in the supply chain including green purchasing, green manufacturing, green material management, green distribution, green marketing, and reverse logistics. It also considers waste reduction in all stages of the supply chain and involves cradle-to-grave product management in supply chain management [22–24]. Product recovery and end-of-life product management have been highlighted in the literature of reverse supply chains and closed loop supply chains. A reverse supply chain involves backward flows of product returns from customer to source [25]. These product returns can be recovered and re-entered in the forward supply chain. The term closed loop supply chain is applied to a chain that consists of both reverse and forward supply chains [17, 26].

Based on the literature review conducted for this research, the term sustainability entered the supply chain literature after 2001. To date, apart from a few exceptions, the contents of publications are still limited to environmentally conscious issues. However, some authors (e.g., [17, 27, 28]) have tried to define the term sustainable supply chain management (SSCM). They have integrated the definition of supply chain management with the triple bottom line of sustainability that consists of environmental, social, and economic performance. For example, Seuring and Müller [28, pp. 1700] defined SSCM as “*the management of material, information and capital flows as well as cooperation among companies along the supply chain while taking goals from all three dimensions of sustainable development, that is, economic, environmental and social, into account which are derived from customer and stakeholder requirements.*”

Taking into account the above-mentioned definition of a SSC and by integrating the concept of the closed loop supply chain, green supply chain, and organizational sustainability, a SSC can be summarized as *“a supply chain that closes the loop of upstream and downstream flows of products and materials by recycling and recovering used-items and re-entering them in production cycles and engages in sustainability-conscious practices taking goals from all three dimensions*—*economic, environmental and social*—*of sustainable development into account, which are derived from the customer and stakeholder requirements.”*  As a result, SSC practices not only positively affect the natural environment and society, but also result in long-term economic benefits and competitive advantage for the entire chain.

## 3. Research Process

The research process of this study is divided into three stages. The first stage is to understand the components of SSCD. Secondly, the SSC practices, processes, and structures are categorized accordingly. Finally, the framework for configuring a SSC is developed. These research stages are presented in the subsequent subsections.

### 3.1. Understanding the Components of SSCD

A comprehensive literature review was conducted on the credible literature published within the last 18 years to identify and understand the components of SSCD in terms of practices, processes, and structures. A combination of keywords, such as supply chain/logistics, sustainable, green, environment, reverse, closed-loop, waste, return, reuse, recycle, remanufacture, and product recovery, were used in the literature search. These keywords were shortlisted based on those used by authors of highly cited research papers.

The list of keywords was used to search for articles from 1995 to 2012 on the Web of Science database to cover a significant proportion of the credible and reliable literature. The initial search resulted in 3,274 research articles and 109 review papers. The search results were refined by keeping engineering, management, business, and environmental science categories and excluding other categories.

The list was then reduced to 341 papers after analysis of the titles and abstracts and selection of only the highly related articles. Each paper was then assigned to one or multiple components of SSCD based on the content of the article. Several papers were also added based on bibliographical analysis and some were rejected due to irrelevancy. The final list, based on both journal papers and bibliographic search, was refined to 309 articles to which this study will make reference.  Table 2 shows the distribution of articles referred to in this study from various journals within the last 18 years (1995–2012).

### 3.2. Categorizing SSC Practices, Processes, and Structures

This study, by reviewing the literature, has attempted to explore the different processes required to manage a SSC. The Supply Chain Operation Reference or SCOR model [29] was used as a master guide to categorize and define these processes. SCOR is a well-known model in the literature pertaining to supply chains developed by the Supply Chain Council.

SSC structures in terms of forward and reverse networks should be designed to implement SSC processes. This study, by reviewing the literature, presents a classification of the various types of network.

A combination of IDEF0 (a function modelling method) and IDEF3 (a process modelling method) [30–33] was used to classify the SSC practices found in the literature. The process-oriented approach involved in these methods provides the basis for harmonizing the SSC practices with the SSC processes and structures.

### 3.3. Developing the Framework for Configuring a SSC

In the final stage, a conceptual framework is developed for configuring the SSC practices, processes, and structures. In accordance with the configurational approach used in this stage, a central theme is required to harmonize the various components of a SSC. The concept presented in NRBV [14, 15] is adapted to determine this central theme. According to NRBV, implementing different environmental strategies can generate different kinds of value for stakeholders. These values are cost and risk reduction, reputation and legitimacy, and innovation and repositioning. These three categorizations of values can be considered as central themes forming three types of configuration for SSCs.

## 4. SSC Processes, Structures, and Practices

### 4.1. SSC Processes

The Supply Chain Operation Reference model (SCOR) [29] was used as a master guide to determine the main subprocesses in SSC. SCOR is a well-known model in the literature of supply chains developed by the Supply Chain Council. The main processes in this model are plan, source, make, deliver, and return.

**Figure 1:**
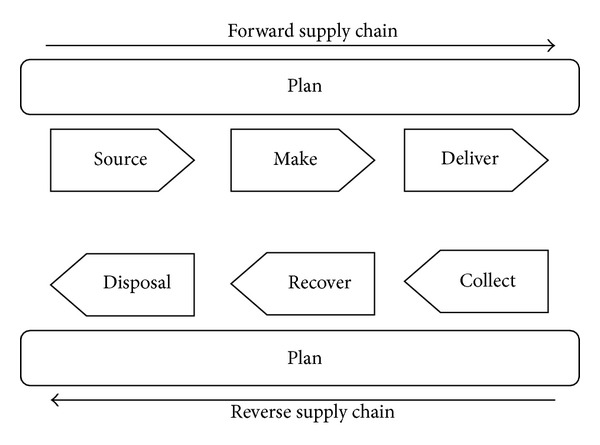
Sustainable supply chain processes: main categorization (adapted from SCOR model [29]).

From the definitions of the “plan” process in the SCOR model, four main subprocesses are derived from the SSC literature, namely, inventory control and management, production and capacity management, green supplier management, and green marketing. In respect of the importance of the “return” process in SSC, the process is extended to three sub-processes, namely, collect, recover, and dispose.  Figure 1 illustrates the main processes of a SSC.  Table 2 also depicts the definitions offered by the authors for these processes. The definitions provided by the Supply Chain Council [29] are considered in the authors' definitions and are customized based on the SSC literature. To better understand the definitions, the related literature on each process is also presented in Table 3.

### 4.2. SSC Structures

Different organizations or individuals are required to play a role in a supply chain in order to implement the SSC processes. From the literature [34–37], there are five types of network through which the processes of forward and reverse supply chains are accomplished:

- forward supply chain networks, which include suppliers, producers, and distributors that are designed for producing original products and distributing them to the market;
- collection networks (CO), which are designed for collecting used products from consumers and distributing them to relevant destination points based on feasible recovery options;
- reprocessing networks (REP) that transform used products to reusable products through repairing, refurbishing, remanufacturing or cannibalizing, and distributing them to the reuse market;
- recycling networks (REC), which extract reusable materials from used products and distribute them to suppliers;
- waste treatment networks (WT) that direct unusable products and materials to disposal or landfill sites.

**Figure 2:**
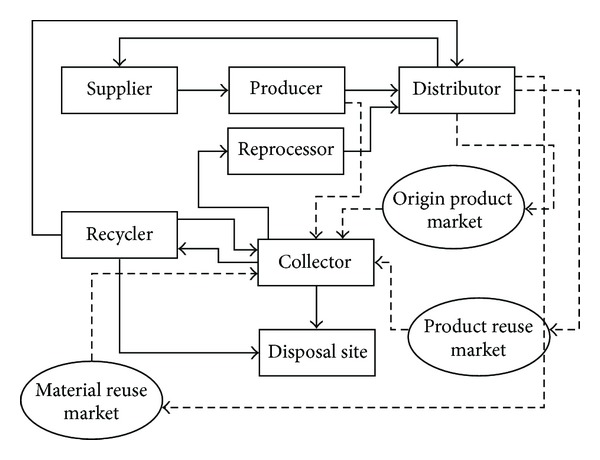
A typical structure for sustainable supply chain (adapted from Fleischmann et al. [34], Mutha and Pokharel [36], Olugu et al. [37], and Sheu et al. [35]).

As can be seen from Figure 2, in the forward supply chain, raw materials are taken from suppliers and pass through the “make” process undertaken by a manufacturer who is the producer. The final products produced in the forward supply chain are delivered to customers and end-users by a distributor. Later on, through the reverse chain, used products are collected by a collector and separated into recoverable or unusable units. Unusable products are carried to disposal sites and recoverable products are transferred either to a recycler or to a reprocessor, according to the processes required for their recovery. Recycled materials would be re-entered into the forward chain or sold to the market. Reprocessed products can also be sold in the used-products market. The waste produced in the “make” process is also transferred to a collector in the reverse supply chain, where they would be transferred to a disposal site or passed to a re-processor or recycler according to the content of the produced waste.

### 4.3. SSC Practices

**Figure 3:**
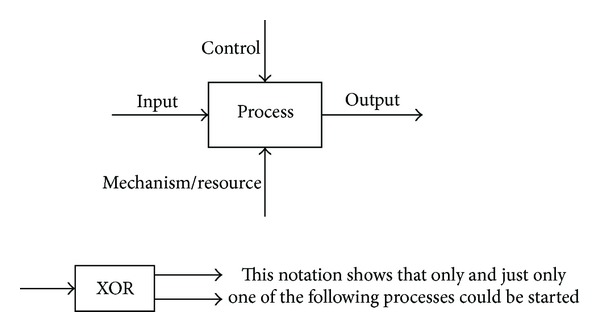
IDEF0 and IDEF3 notations [31] used in this paper.

Different practices in managing environmentally conscious supply chains are identified from the collected literature. These practices are classified under nine headings using IDEF0 and IDEF3 [30–33], which are the function and process modelling methods, respectively.  Figure 3 illustrates the notations of these methods, as used in this paper.

**Figure 4:**
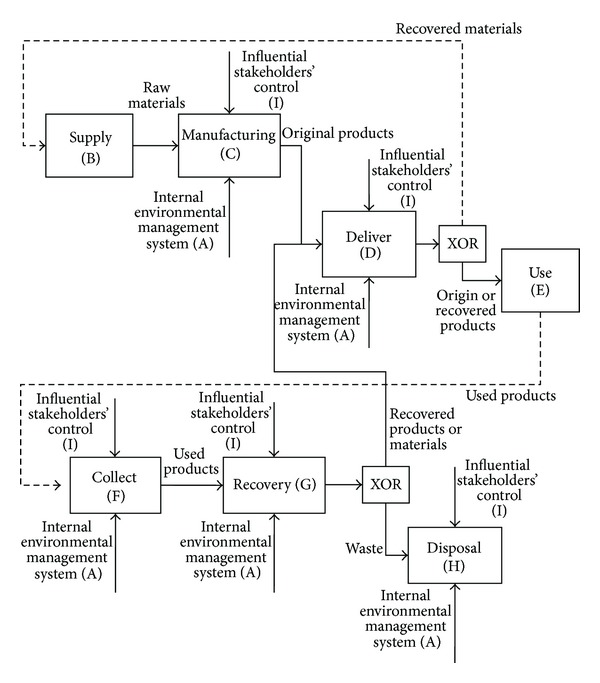
A process-oriented approach for categorizing sustainable supply chain practices.

Figure 4 shows the process model of a typical green supply chain, which classifies green supply chain practices into nine groups. The practices labelled from group B to H are related to the green supply chain processes. Group A practices refer to the mechanism used to implement these processes while Group I practices relate to managing the standards and regulations imposed by stakeholders to control the processes.  Table 4 presents the sample practices for each group as found in the literature.

#### 4.3.1. Group A: Internal Environmental Management

To implement green supply chain practices, the organizations are required to establish their own environmental management system. These systems provide the mechanism for implementing green supply chain practices and will have a remarkable impact on the firm's environmental performance [38]. Management commitment and support for green supply chain initiatives are also key measures for success in greening the supply chains [39].

#### 4.3.2. Group B: Green Supply and Supplier Management

Practices that relate to the supply of raw materials and components are categorized in this group. There are two main themes for green supply.

The first theme is to utilize the used products and recycled materials as a valuable source of components and materials [40–42]. Proactive companies would try to increase the cost of OEM returns' acquisition for their competitors and create a competitive advantage for themselves [43]. However, the implementation of these practices requires new technologies to recover and reuse the materials [22].

The second theme is to establish a green purchasing procedure by making relevant changes in raw materials and utilization of environmentally friendly materials [44], green supplier selection and green supplier relationship management [41, 44–47], integrating purchasing strategies with product recovery strategies [48] and involving components suppliers in product design [40].

The first theme of utilizing used products is known as the waste-directed approach, whereas the second theme of making changes in raw materials and use of environmentally friendly materials is known as the source-directed approach [22]. In order to adopt these approaches, companies are required to collaborate with their suppliers concerning the environmental aspects [49–51].

#### 4.3.3. Group C: Green Manufacturing

The majority of practices in this group involve the waste-directed approach [22]. The main purpose of these practices is to increase product recovery feasibility and the values created by product recovery management. Some examples of these practices are product eco-design for product recovery [40, 52] and process redesign in order to reduce solid waste [44].

#### 4.3.4. Group D: Green Deliver

The practices categorized in this group are those that minimize the environmental impact of products' delivered in terms of transportation and packaging. For example, companies can employ a more environmentally friendly transportation method [44] and use recyclable or reusable packaging [53] to decrease the burden of the products delivery operations on the environment.

#### 4.3.5. Group E: Green Consumption and Customer Management

This group of practices follows two main objectives. The first objective is to promote a green image and encourage the customers to purchase green or recovered products and components. The second objective is to provide the customers with insights into green consumption so that they could use products efficiently and reduce any related environmental impact during the stage of consumption.

Green customer management practices intend to promote green products into the market via various different strategies. For example, recovered products could be sold as new products or be resold as used products but at a lower price [54]. Recovered components and materials of used products could also be used internally by the company itself or sold to the market within or outside the business chain [40]. Some authors emphasized green marketing and managing the customers' perception of quality to promote a green image [40, 44, 55–57]. Collaboration with customers for environmentally conscious practices [39] can also be considered as an effective practice to promote a green image.

#### 4.3.6. Group F: Collection Management

Collecting used products from customers and consumers is an important strategic issue in a SSC. To close the supply chain loop, it is necessary to collect and recover used products. The main purpose of these practices is to increase the rate of collection for used products while considering the economic and environmental impacts. There are many different methods to collect used products, such as taking back products by law or contract, returning products by off-lease or off-rent contracts [40].

#### 4.3.7. Group G: Recovery Management

The main objective of the practices within this group is to minimize the generated waste by recovering the used-products in terms of repairing, refurbishing and remanufacturing, or recycling materials whenever possible [40].

#### 4.3.8. Group H: Waste Disposal

The waste disposal practices originated from the effect-directed approach, which is a less integrated approach to manage an environmentally conscious supply chain [22]. Some examples of practices categorized in this group are cooperation with waste management companies [40] and proper disposal of hazardous materials/chemicals/equipment.

#### 4.3.9. Group I: Influential Stakeholder Management

Practices categorized under this group aim to minimize the cost of meeting the regulators' requirements and to increase the company's flexibility and responsiveness by influencing the key stockholders. The central theme of these initiatives is to leverage on co-operating practices. For instance, involvement of a large number of companies in the process of environment-related legislation could influence the draft for the new legislation [40, 41]. Another example of practices under this group is cooperation with stockholders of the remanufacturing supply chain [56].

## 5. A Conceptual Framework for Configuring SSC Practices, Processes, and Structures

The design components of a SSC including SSC practices, processes, and structures have been identified and described in the previous section. The process-oriented approach applied to categorize SSC practices could be considered as an initial effort to configure these components.  Table 5 demonstrates the match between the SSC practices, processes, and structures at a glance.

From a configurational approach, a central theme is required to be created in order to harmonize the design components. Miller [7] considered different kinds of competitive strategy to create this central theme and suggested different strategic configurations. As it is important to create long-term economic benefit and competitive advantage for a SSC, this study adopted Miller's [7] approach to create different configurations of a SSC. Also, for this purpose, this study also refers to the NRBV [14, 15] to understand how the environmental strategies could provide sustainable value for shareholders, and, consequently, competitive advantage for the firms.

NRBV [14, 15] states that there are interconnected strategies to address environmental drivers, namely, “*pollution prevention*”, “*clean technology*”, and “*product stewardship*”, which can provide sustainable value for shareholders in terms of “*risk and cost reduction*”, “*innovation and repositioning*”, and “*reputation and legitimacy*”, respectively. Considering these three kinds of environmental strategy, three configurations for SSC can be organized: efficient SSC adopting pollution prevention strategy; innovative SSC adopting clean technology strategy; and reputed SSC adopting product stewardship strategy. These configurations are further explained in the following paragraphs.

*Efficient SSCs.* Is the first type of SSC configuration, which can be used by companies adopting the “pollution prevention” strategy in order to reduce their cost and risk. They aim to reduce, change, or prevent the emissions by involving practices, such as material substitution, recycling of materials internally in the company, and process innovation [14, 15]. This approach, also known as waste-directed or emission-directed, is a more integrated approach compared to the effect-directed approach, which only deals with waste disposal issues [22]. According to [14], this strategy can build new capabilities in operations and develop the key resource of continuous improvement to provide *cost and risk reduction advantages* for the firms.

*Innovative SSC.* Is the second type of SSC configuration that can be used by companies adopting a “clean technology” strategy. These companies search for innovative solutions to tackle environmental problems and sustainable supply challenges by depending on more sustainable and clean technologies. Clean technologies can provide the opportunities for the organizations to reposition their internal skills and capabilities to gain benefits from future markets. Innovation and repositioning are the values that firms would propose to their shareholders through the implementation of this kind of environmental strategy [15].

*Reputed SSC.* Is the third type of SSC configuration that can be used by the companies adopting a “product stewardship” strategy. These companies attempt to integrate different stakeholder's views into the business processes to provide reputation and legitimacy for the firm. Product stewardship, which involves the whole chain from raw materials to the disposal of generated waste, is a more integrated approach compared to pollution prevention. Some practices that organizations can consider to create sustainable value to their shareholders through this strategy are green marketing efforts relating customers' purchasing actions to sustainability conscious decisions; life cycle management considering the costs and benefits of products beyond the internal boundaries of the firms (from the sources of materials to disposal of the ultimate waste by end-users); and closing the supply chain loop by converting the wastes into new inputs and re-entering the used materials and products in the production cycle [14, 15]. By implementing the product stewardship strategy, firms might gain the competitive advantage of being the first mover in future markets. This can be the result of acquiring limited resources for producing green products or establishing a set of new and tailored rules and regulations in interaction with influential stakeholders [14].

**Figure 5:**
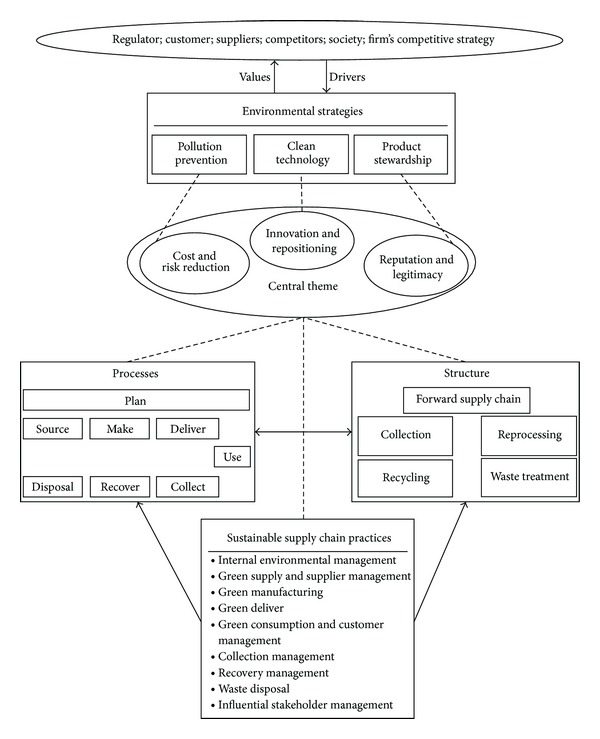
A conceptual framework for designing a sustainable supply chain.

As can be seen from Figure 5, the drivers from various stakeholders influence the companies' decisions for selecting the appropriate environmental strategy. The consequent values expected to be created by this strategy can be considered as a central theme for configuring the SSC. These drivers can either be external or internal. Examples of external drivers are regulators, customers, suppliers, green associations, NGOs, and competitors that drive the company to implement green practices to meet their expectations [58, 59]. Internal drivers [58, 59] could be the firm's environmental mission and competitive strategy which motivate the company to seek for environmentally friendly solutions that not only meet the external stakeholders' requirements, but also improve the firm's competitiveness.

After selecting the dominant environmental strategy, the appropriate configuration of SSC practices, processes, and structures can be designed. Firstly, the core practices should be determined by considering the central theme. Secondly, the processes and structures should be designed to implement these practices while simultaneously following the central theme.

Table 4 shows the recommended matching of processes and networks with the appropriate SSC practices.  Table 6 briefly describes how these processes and networks should be designed to configure each of the three above-mentioned configurations. These recommendations for core practices, processes, and networks are based on the central theme and philosophy of each kind of environmental strategy, as presented in this section.

## 6. Conclusion and Research Implications

Pressure from different stakeholders to integrate sustainability conscious aspects in business practices has driven enterprises to adopt a variety of green initiatives in their supply chain. It goes without saying that no business can address all of these practices due to resource and budget limitations [60]. Therefore, they have to make a decision in selecting the most strategic practices for their business, and, subsequently, provide the appropriate infrastructure for implementing such practices. In other words, the company has to decide on the desired values created by the implementation of these practices and then design the whole sustainable supply chain (SSC) to offer these values as much as possible. By considering this requirement, this study has embarked on a process-oriented approach to produce a comprehensive list of SSC practices classified into nine groups, namely, internal environmental management, green supply and supplier management, green manufacturing, green deliver, green consumption and customer management, collection management, recovery management, waste disposal, and influential stakeholder management. The process-oriented approach to classify these practices provides a basis for matching the practices to the processes and structures. The main processes are plan, source, make, deliver, use, collect, recover, and waste disposal. The structures include both forward and reverse supply chain networks in which the reverse networks are categorized into four distinctive structures: the waste treatment, collection, reprocessing, and recycling networks.

Afterwards, by applying a configuration approach [7, 8], three kinds of SSC configuration are suggested, namely, efficient SSC, innovative SSC, and reputed SSC. These configurations are developed based on the philosophy of various environmental strategies proposed by [14, 15] with an emphasis on the natural-resourced-based view (NRBV).

Efficient SSCs follow the pollution prevention [14, 15] strategy, which intends to minimize the waste and emissions from the operations. The central theme for this configuration is cost and risk reduction. Core practices to meet the requirements of this environmental strategy could be green supply and purchasing, green process design, and material, product, and investment recovery. The processes would be designed to be costeffective, thus they are usually standardised and procedural. A centralized design of structures [34] might lead to cost reduction throughout the whole chain.

Innovative SSCs follow the clean technology strategy [15], which intends to develop the competencies for innovative development and future shaping. The central theme for this configuration is innovation and repositioning. Product eco-design and use of clean energy and technology are the core practices to meet this configuration's requirements. Processes are usually flexible and innovative to provide the rapid development of competencies required for the future. Decentralized structures [34] could also be considered as a solution for designing the reverse networks to provide a basis for rapid development.

Reputable SSCs follow the product stewardship strategy [14, 15], which intends to integrate stakeholder views into the business process. The central theme for this configuration is reputation and legitimacy. Collaborative practices in terms of collaboration with suppliers and customers and involving the influential stakeholders in business practices can be considered as core practices in this configuration. Processes should also be designed for effective collaboration with influential stakeholders. Reverse networks in this kind of configuration can also be developed by a joint approach through an alliance with existing reputable networks in the industry. While designing the networks for this configuration, closing the loop is also a critical measure.

This study has applied a process-oriented approach to classify SSC practices in addition to a configurational approach for configuring these practices with the processes and structure. It forms the initial efforts in developing a framework for sustainable supply chain design (SSCD). By configuring and harmonizing the design components of a SSC, this framework could assist companies to gain more benefit from implementing sustainability conscious practices. Future research for validating these configurations, such as conducting a series of case studies involving organizations from various industries, would develop this field of research further.

Moreover, future studies for exploring the particular SSC practices in various industries and customizing the list of SSC practices for each unique combination of industries and configurations might develop the field further.

Once the framework is validated, the measurement methods can be developed to determine the degree of alignment of existing configurations to that of the standard configuration presented in the framework. Finally, this measurement method would provide a quantitative research framework for analysing the link between an appropriately configured SSC and the firm's performance and competitiveness.

## Figures and Tables

**Table 1:** The list of previous studies in sustainable supply chain practices, processes, and structures.

Area	References
Sustainable supply chain practices, drivers, and performances	[39, 44, 58, 59, 61–72]
Optimizing planning processes	[73–86]
Network design	[34, 36, 87–102]

**Table 2:** The distribution of articles in various journals over the last 18 years.

Journal	Numbers of articles
International Journal of Production Research	17
Journal of Cleaner Production	13
European Journal of Operational Research	10
International Journal of Production Economics	7
Computers and Operations Research	6
Supply Chain Management-an international journal	6
Resources Conservation and Recycling	5
Computers and Industrial Engineering	4
Journal of Operations Management	4
Harvard Business Review	3
International Journal of Environmental Science and Technology	3
International Journal of Operations and Production Management	3
California Management Review	2
International Journal of Advanced Manufacturing Technology	2
International Journal of Physical Distribution and Logistics Management	2
Journal of Business Logistics	2
Journal of Environmental Management	2
Omega-International Journal of Management Science	2
Production and operations management	2
Production planning and control	2
Transportation Research Part E-Logistics and Transportation Review	2
Academy of Management Journal	1
Academy of management review	1
Benchmarking: an international journal	1
Business Strategy and the Environment	1
Computers in Industry	1
Environmental Health Perspectives	1
Industrial Management and Data Systems	1
Interfaces	1
International Journal of Logistics Management	1
International Journal of Management Reviews	1
Journal of Management Studies	1
Journal of Mechanical Design	1
Journal of the Operational Research Society	1
Logistics Information Management	1
M and Som-Manufacturing and Service Operations Management	1
Management Science	1
Mathematical and Computer Modelling	1
Strategic Management Journal	1
Technovation	1
The Academy of Management Executive (1993–2005)	1
The International Journal of Logistics Management	1
**Total**	**118**

**Table 3:** Sustainable supply chain processes and their definitions.

SSC Process	Definition	Related literature
Plan		
Inventory control and management	Processes that manage inventory regarding the high variability and uncertainty in time, quality and quantity of returns, and demand for recovered materials.	[103–110]
Production and capacity management	Processes that plan production and manage the capacities of manufacturers and recovery centres to achieve a balance between demand and returns considering the high degree of uncertainty and complexity in recovery management systems.	[75, 81, 84, 103]
Green supplier management	Processes that select and evaluate suppliers considering environmental issues; cooperate with them to enhance environmental performance and promote the initiatives and practices for greening the suppliers.	[45, 46, 108, 111–113]
Green marketing	Processes that promote a green image and persuade customers that the green and environmentally friendly products meet their requirements.	[114, 115]
Source	Processes that procure materials and components that have a lower impact on the environment and also consider recovered products as a valuable source of materials and components.	[40, 116]
Make	Processes that produce products that have a lower impact on the environment.	[117, 118]
Deliver	Processes that provide both original and recovered products to meet the uncertain demand, including order management, environmentally friendly packaging, and sustainable transportation systems.	[119, 120]
Return		
Collect	Processes that involve the collection operations of returned products, inspection, and separation of recoverable products from disposal and delivering the recoverable products to related places.	[34, 117]
Recover	Processes that transform returned and used products to reusable products, typically including repairing, refurbishing, remanufacturing, cannibalizing, and recycling.	[34, 119]
Dispose	Processes that include land filling or incinerating products that are rejected at the “Collect” process.	[40, 117]

**Table 4:** Sustainable supply chain practices presented in the literature.

A: internal environmental management (1) Commitment for GSCM from senior managers [39]	
(2) Support for GSCM from mid-level managers [39]	
(3) Cross-functional cooperation for environmental improvements [39]	
(4) Total quality environmental management [39]	
(5) ISO 14000 certification [39]	
(6) Environmental compliance and auditing programmes [39]	
(7) Environmental management systems exist [39]	
B: green supply and supplier management	
*B1: green supply and purchasing *	
(8) Use of used products as a valuable source of components and materials [40]	
(9) Use of environmentally friendly raw materials [44]	
(10) Substitution of polluting and hazardous materials/parts [44, 53]	
(11) Use of the company waste of others [44]	
(12) Providing design specification to suppliers that include environmental requirements for purchased item [49]	
(13) Supplier selection involving environmental criteria [44]	
(14) Urging suppliers to establish environmental management systems [44]	
*B2: supplier environmental collaboration *	
(15) Providing suppliers with educational, technical, and financial support to establish and implement their own environmental programme [44, 121]	
(16) Holding awareness seminars for suppliers on environmentally conscious actions and their benefits [44]	
(17) Facilitating sharing knowledge and lessons learned relating to environmental issues between different suppliers [44]	
(18) Collaboration with suppliers to provide materials, equipment, parts, and services that support environmental goals [49]	
(19) Involving component suppliers in product design [40]	
(20) Environmental audit of suppliers' internal management [49]	
(21) Second-tier supplier environmentally friendly practice evaluation [39] Joint long-term programmes to develop green innovations and solutions [121]	
C: green manufacturing	
*C1: product eco-design *	
(22) Design of products for reduced consumption of material and energy [49]	
(23) Design of products to reduce or avoid pollution and waste generation in product usage and/or in their manufacturing process [49]	
(24) Design of products to avoid or reduce the use of hazardous materials in products/or their manufacturing process [49]	
(25) Design of products for reuse, recycling, recovery of materials, components, and parts [49]	
(26) Design of products for remanufacturing, repair, rework, and refurbishing activities [19]	
(27) Product design considering product life cycle costs [56]	
*C2: green process design *	
(28) Optimization of production planning and manufacturing processes to reduce waste and optimize material exploitation [44]	
(29) Optimization of manufacturing processes to reduce energy and natural resource consumption [44]	
(30) Optimization of manufacturing processes to reduce solid and water waste, and air emissions [44]	
(31) Optimization of manufacturing processes to reduce noise pollution [44]	
*C3: use of clean energy and technology *	
(32) Use of clean technology to make savings [44]	
(33) Use of clean sources of energy [44]	
D: green deliver	
*D1: green distribution and transportation *	
(34) Use of more environmentally friendly transportation method [44]	
*D2: green packaging *	
(35) Use of recyclable or reusable packaging/containers in logistics [53]	
(36) Use of ecological materials for primary packaging [53]	
E: green consumption and customer management	
*E1: green consumption management *	
(37) Eco-labelling of products [44]	
(38) Green marketing and managing customer's perception of quality to promote green image [40]	
(39) Environmental pricing to promote extended product responsibility [122]	
(40) Providing consumers with information on environmental friendly products and/or production methods [44]	
(41) Providing instructions for environmentally friendly use of products [22]	
*E2: customer environmental collaboration *	
(42) Cooperation with customer for eco-design [39]	
(43) Cooperation with customer for cleaner production [39]	
(44) Cooperation with customer for green packaging [39]	
F: collection management	
(45) To collect used products in an effective way (directly from customers or from used products broker, directly by companies or by a retailer/third service provider) in order to facilitate collection activities and to increase the amount of used products' return [22, 40, 55, 123]	
(46) To take back products by law or by contract [22, 40]	
(47) Buyback pricing with regard to the targeted amount for collecting and the price of competitors [124]	
G: recovery management	
*G1: material recovery *	
(48) Internal recycling of materials within production phase [44]	
(49) Taking back packaging [44]	
(50) Labelling material packages for retrieval purposes [53]	
*G2: product recovery *	
(51) Recovery of the company's end-of-life products [44]	
(52) Recover products whenever possible and choose the product recovery and disposition options based on product characteristics and technical feasibility, supply of components and materials, demand for recovered products and economical and environmental impacts [40]	
*G3: investment recovery *	
(53) Sale of excess inventories/materials [49]	
(54) Sale of scrap and used materials [49]	
(55) Sale of excess capital equipment [49]	
H: waste disposal	
(56) Disposal of hazardous materials/chemicals/equipment [49]	
(57) Cooperation with waste-management companies [40]	
I: influential stakeholder management	
(58) Publicizing environmental efforts, promoting industry cooperative efforts and collaboration [41]	
(59) To manage the competitors by imposing a set of private regulations or by shaping the governmental rules [125]	

**Table 5:** A harmonized categorization of sustainable supply chain practice, processes, and structures.

Practices	Processes	Structures
Internal environmental management	Mechanisms and systems for implementing processes	
Green supply and purchasing	Source	Forward supply chain
Supplier environmental collaboration	Source	Forward supply chain
Product eco-design	Make	Forward supply chain
Green process design	Make	Forward supply chain
Use of clean energy and technology	Make	Forward supply chain
Green distribution and transportation	Deliver	Forward supply chain
Green packaging	Deliver	Forward supply chain
Green consumption management	Use	Forward supply chain
Customer environmental collaboration	Use	Forward supply chain
Collection management	Collect	Collection networks
Material recovery	Recover	Recycling networks
Product recovery	Recover	Reprocessing networks
Investment recovery	Recover	Collection networks
Waste disposal	Disposal	Waste disposal networks
Influential stakeholder management	Managing the external drivers/regulations that control the processes	

**Table 6:** Different configurations of a sustainable supply chain.

	Efficient SSC	Innovative SSC	Reputed SSC
Central theme	Cost and risk reduction [15]	Innovation and repositioning [15]	Reputation & legitimacy [15]
Dominant environmental strategy	Pollution prevention [15]	Clean technology [15]	Product stewardship [15]
Philosophy	Minimize waste and emissions from operations [15]	Develop the sustainable competencies of the future [15]	Integrate stakeholders views into business process [15]
Core practices	(i) Green supply and purchasing (ii) Green process design	(i) Product eco-design (ii) Use of clean energy and technology	(i) Supplier environmental collaboration (ii) Customer environmental collaboration (iii) Influential stakeholder management (iv) Material & product, recovery
Processes	Standard and procedural	Innovative and fast response	Collaborative
Networks	Centralized [34]	Decentralized [34]	Joint venture or alliance with reputed networks in industry [34] Closed loop [15, 34]
